# Mycobacterium tuberculosis Rv1324 Protein Contributes to Mycobacterial Persistence and Causes Pathological Lung Injury in Mice by Inducing Ferroptosis

**DOI:** 10.1128/spectrum.02526-22

**Published:** 2023-01-10

**Authors:** Xiaoxia Shi, Chunyu Li, Lin Cheng, Hayan Ullah, Shanshan Sha, Jian Kang, Xiaochi Ma, Yufang Ma

**Affiliations:** a Department of Biochemistry and Molecular Biology, Dalian Medical University, Dalian, China; b College of Integrative Medicine, Dalian Medical University, Dalian, China; c Department of Experimental Teaching Center of Public Health, Dalian Medical University, Dalian, China; d Department of Microbiology, Dalian Medical University, Dalian, China; e Pharmaceutical Research Center, The Second Affiliated Hospital, Dalian Medical University, Dalian, China; Indian Institute of Science Bangalore

**Keywords:** *Mycobacterium tuberculosis*, Rv1324, oxidative stress, ferroptosis, pathogenesis

## Abstract

Mycobacterium tuberculosis (Mtb) is the pathogenic agent of tuberculosis (TB). Intracellular survival plays a central role in the pathogenesis of Mtb, a process that depends on an array of virulence factors for Mtb to colonize and proliferate within a host. Reactive nitrogen and oxygen species (RNS and ROS) are among the most effective antimycobacterial molecules generated by the host during infection. However, Mtb has evolved a number of proteins and enzymes to detoxify ROS and RNS. Secretory protein Rv1324, as a possible thioredoxin, might also have oxidoreductase activity against ROS and RNS during Mtb infection, and it is a potential virulence factor of Mtb. In this study, we investigated the biochemical properties of Mtb Rv1324 and its role in mycobacterial survival and virulence. The results showed that the Rv1324 protein had antioxidant activity and increased the survival of M. smegmatis that was exposed to ROS and RNS. In addition, Rv1324 enhanced the colonization ability of M. smegmatis in the lungs of mice. Further, mice infected with M. smegmatis harboring Rv1324 exhibited pathological injury and inflammation in the lung, which was mediated by ferroptosis. In summary, this study advances our understanding of the mechanisms of mycobacterial survival and pathogenesis, and it reveals a novel target for TB treatment.

**IMPORTANCE** The intracellular survival of M. tuberculosis (Mtb) plays a crucial role in its pathogenesis, which depends on various Mtb oxidoreductases that are resistant to reactive oxygen and nitrogen species (ROS and RNS) that are generated by the host during Mtb infection. Secretory protein Rv1324 is a potential virulence factor of Mtb and is a possible thioredoxin that has oxidoreductase activity against ROS and RNS during Mtb infection. We investigated the biochemical properties of Mtb Rv1324 and its role in mycobacterial survival and virulence. It was confirmed that the Rv1324 protein had antioxidant activity and an increased mycobacterial resistance to ROS and RNS. In addition, Rv1324 enhanced mycobacterial persistence and induced pathological injury and inflammation in the lungs of mice by activating ferroptosis. This study advances our understanding of the mechanisms of mycobacterial survival and pathogenesis, and it reveals a novel target for TB treatment.

## INTRODUCTION

Tuberculosis (TB), which is caused by the pathogenic agent Mycobacterium tuberculosis (Mtb), is a major threat to public health. Globally, one-third of people are estimated to be latently infected with Mtb. The World Health Organization (WHO) reported that an estimated 10.6 million people fell ill with TB and that more than 1.58 million people died from TB in 2021 (1). TB was reported as the disease with the highest fatality rate that was caused by a single pathogen infection before the COVID-19 pandemic. The problem has been further compounded by the emergence of drug-resistant forms, HIV coinfections, diabetes, and the lack of an effective vaccine and diagnostic tools for the rapid detection of TB (1).

Mtb infection is difficult to control. It has been reported that Mtb can escape during the process of infection and can remain dormant in old lesions (1), which is caused by host defense mechanisms that are extensively modulated by Mtb to avoid host immune clearance (2). The early interactions between Mtb and host innate immune cells determine the establishment of the Mtb infection and the development of TB disease (2). Macrophages, as a first-line defensive patrol, quickly respond to Mtb infection and induce host anti-Mtb immunity, such as phagocytosis and apoptosis (3). However, Mtb has a strategy of inhibiting apoptosis (4) and promoting necrosis. It was reported that many Mtb secretory proteins, including ESAT-6, CpnT, and Rv2626c, are necrosis inducers in macrophages (5) and important virulence factors of Mtb. Necrosis results in the release of mycobacteria into the extracellular environment (5), which facilitates the spread of bacteria within the host. Therefore, Mtb has the ability to regulate the death modes of infected cells as an Mtb survival strategy in the host. Recently, ferroptosis, a new type of programmed cell death, was also reported as one kind of mechanism by which Mtb survives and spreads in the host (6). It has been confirmed that macrophages infected by Mtb promote labile iron accumulation and lipid peroxidation. In addition, macrophages that are dying of necrosis have high oxidized lipid contents and low expression of the GPX4 enzyme, which is involved in lipid peroxide removal (7). Macrophage ferroptosis induced by Mtb infection activates a proinflammatory response and drives the necrotic death and rupture of macrophages (6, 7). Eventually, Mtb is released into the extracellular environment and spreads to other cells. Therefore, these studies suggest that ferroptosis is a virulence mechanism that is driven by Mtb.

Free radicals, reactive oxygen species (ROS), and reactive nitrogen species (RNS), are considered to be important signals that trigger cell ferroptosis (8). Generally, the production of ROS is mediated by the Fenton reaction or by the oxidation of iron-binding enzymes, and ROS subsequently triggers lipid peroxidation. Then, the products of lipid peroxidation mediate membrane damage and lead to cell ferroptotic death (8–10). In this process, ROS and RNS induce macrophage ferroptosis and kill intracellular Mtb (11, 12). Thus, neutralizing ROS and RNS is essential for the survival and spread of Mtb in the host. Several studies have indicated that Mtb has several enzymes and proteins that work against the redox stresses of the host. Mtb detoxifies ROS and RNS by activating the expression of genes encoding catalases, peroxidases (13, 14), superoxide dismutases (15), and thioredoxins (Trxs) (16). These protective enzymes and proteins not only help Mtb survive under the redox stresses manifested by the host but are also virulence factors of Mtb (14–19). For example, the secretory proteins SodC (15) and TrxC (16) anchor in the membrane to protect Mtb from ROS and RNS at the bacterial surface and increase the survival and virulence of Mtb in macrophages. Therefore, secretory proteins against ROS and RNS deserve attention, as they are important virulence factors for the survival and virulence of Mtb in the host.

Mtb contains three Trxs, namely, TrxA, TrxB, and TrxC, and these are protein disulfide oxidoreductases. All Trxs possess the conserved “Cys-X-X-Cys” active site in the Trx fold, which catalyzes redox reactions by cycling between a reduced form with dithiol and an oxidized form with a disulfide bond (20). The importance of Trxs in Mtb pathogenesis has been confirmed, and they are involved in resisting oxidative killing by reducing peroxides and dinitrobenzenes (21) and by detoxifying hydroperoxides (22). In particular, TrxC can be secreted from Mtb and may potentially scavenge ROS and RNS in the phagolysosomes of macrophages (23). In addition, the secretory protein Rv1324 has a strong homology with Trxs (24) and may possess a thioredoxin function. One report showed that Rv1324 is a potential novel T cell antigen that is involved in the interaction of Mtb with the host (25). However, the biochemical properties of Rv1324 and its role in the survival and virulence of Mtb have not yet been reported.

In this study, to determine the enzymatic activity of Rv1324, we generated a recombinant E. coli strain, namely, pCold-Rv1324/E. coli BL21(DE3), to overexpress the Rv1324 protein. The purified Rv1324 protein was obtained, and its activity was assessed. To investigate the role of Rv1324 in the survival and virulence of Mtb, a recombinant Mycobacterium smegmatis (M. smegmatis) mc^2^155 strain, namely, Rv1324/Msm, that produces the Rv1324 protein was constructed and used to infect C57BL/6J mice. M. smegmatis mc^2^155 is a model bacterium of Mtb, and it is used to study the effects of Mtb proteins on bacterial pathogenesis (26, 27). The characteristics of Rv1324/Msm were examined in an *in vitro* growth model in response to stresses and in a model of infected mice (26). After infection, the bacterial burden, lung pathology, serum cytokines, and ferroptosis biomarkers of the infected mice were observed or assessed. This study will advance our understanding of the mechanisms of mycobacterial survival and pathogenesis.

## RESULTS

### Generation of the Rv1324/Msm strain.

**FIG 1:**
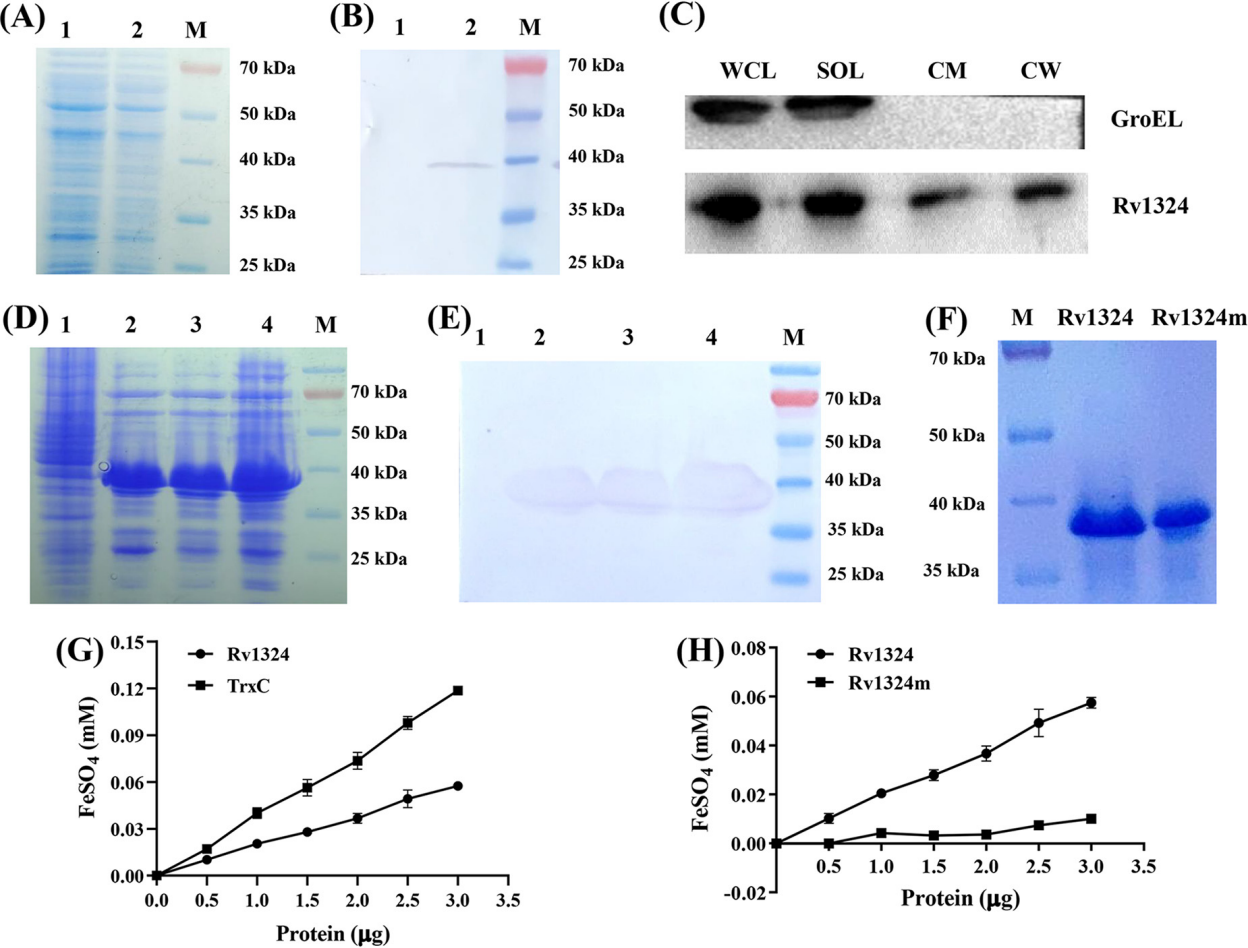
Expression and activity of the recombinant Rv1324 protein. (A and B) The expression of the Rv1324 protein in the Rv1324/Msm strain was analyzed by SDS-PAGE (A) and Western blotting (B). The supernatant of the Vec/Msm and Rv1324/Msm strains were collected. 20 micrograms of the whole-cell lysate proteins of Rv1324/Msm strains were added to the SDS-PAGE gels to analyze the Rv1324 protein expression. For the Western blotting, the proteins bands were visualized in NBT/BCIP solution. The whole-cell lysate proteins of Vec/Msm were used as a control protein. Lane 1 represents the proteins expressed in the supernatant of the Vec/Msm strain. Lane 2 represents the protein expressed in the supernatant of Rv1324/Msm strain number 1. Lane M represents the PageRuler prestained protein ladder. (C) The subcellular localization of the recombinant Rv1324 protein in the Rv1324/Msm strain detected by Western blotting. The proteins bands were visualized using WesternBright ECL detection reagents. WCL represents the whole-cell lysate proteins. CW represents the cell wall fraction. CM represents the cell membrane fraction. SOL represents the soluble fraction of the Rv1324/Msm cells. The expression of the GroEL protein was used as a control protein. (D and E) Analysis of Rv1324 protein expression by SDS-PAGE (D) and Western blotting (E). For the Western blotting, the proteins bands were visualized in NBT/BCIP solution. Lane 1 represents the proteins expressed in the supernatant of E. coli BL21(DE3). Lanes 2 to 4 represent the protein expressed in the supernatant of pCold-Rv1324/E. coli BL21(DE3) strains numbers 1 to 3. Lane M represents the PageRuler prestained protein ladder. (F) SDS-PAGE analysis of the purified recombinant Rv1324 and Rv1324m proteins. (G) Assessment of the antioxidant activity of the recombinant Rv1324 and TrxC proteins. M. tuberculosis TrxC, which was used as a control protein, was also constructed, expressed, and purified using the same protocol with Rv1324. (H) Assessment of the antioxidant activity of the recombinant Rv1324 and Rv1324m proteins.

The mycobacterial expression vector pVV2-Rv1324 was constructed to produce the Rv1324 protein in M. smegmatis cells. Western blotting showed that the recombinant Rv1324 protein band had a molecular mass of approximately 38 kDa (Fig. 1A and B). The predicted molecular mass of the Rv1324 fusion protein with an N-terminal histidine tag was 33 kDa.

Rv1324 is predicted to be located in the cell wall of Mtb. To confirm whether Rv1324 was present in the cell wall and cell membrane fractions of Rv1324/Msm, the expressed recombinant Rv1324 protein in the cell wall, cytoplasm, and cell membrane fractions of Rv1324/Msm was assessed via Western blotting. Although the majority of the Rv1324 protein was detected in the soluble fraction, small amounts were also seen in the cell wall and cell membrane fractions (Fig. 1C), indicating that the Rv1324 protein was transferred to the cell envelope after translation.

### Activity analysis of the Rv1324 protein.

To investigate the activity of the Rv1324 protein, the Rv1324 gene was overexpressed in E. coli cells (Fig. 1D and E), and recombinant Rv1324 protein was purified via Ni^2+^ affinity chromatography. The results showed that a sufficient amount of purified Rv1324 protein was obtained (Fig. 1F). Moreover, the purified protein was analyzed via matrix-assisted laser desorption/ionization time-of-flight mass-spectrometry (MALDI-TOF MS). The identified peptides were well-matched to the peptides of Rv1324, which confirmed that the purified protein was the Rv1324 protein (data shown in Fig. S1).

The amino acid sequences of the three Mtb Trxs and Rv1324 were aligned. The residues in the vicinity of the active Cys site are known to maintain appropriate pKa and redox values (28). The alignment of our sequences showed major changes in the redox-active sites (data shown in Fig. S2). The “Cys-X-X-Cys” motif in TrxA, TrxB, and TrxC (which is conserved in all other bacterial thioredoxins) was replaced with the “Ser-X-X-Cys” motif in the Rv1324 protein. In general, Mtb Trx displayed the ability to reduce disulfide bonds in insulin, a model substrate (23). In this study, we also assessed the ability of Rv1324 to reduce disulfide bonds in insulin. Compared to Mtb TrxC, Rv1324 could not reduce the disulfide bonds in insulin in the presence of DTT (Fig. S3).

Trxs have been demonstrated to possess general intracellular antioxidant activity and protection against oxidative stress (29). We assessed the antioxidant capacity of the Rv1324 protein via a Fe^3+^ reducing power assay. As shown in Fig. 1G, the total antioxidant activity of Rv1324 was significantly enhanced (*P < *0.05) and increased in a dose-dependent manner (Fig. 1G). However, the antioxidant activity of Rv1324 was decreased, compared to that of TrxC (Fig. 1G).

To confirm the antioxidant active site of Rv1324, the Cys_78_ of the Rv1324 protein was replaced by Ala_78_, using a site-directed mutagenesis method. Then, the Rv1324 mutant (Rv1324m) protein was expressed and assessed via SDS-PAGE and Western blotting analyses. As shown in Fig. S4, the Rv1324m protein was overexpressed in E. coli BL21(DE3). Then, the Rv1324m protein was purified, and the purity of both the Rv1324 and Rv1324m proteins was confirmed via a SDS-PAGE analysis (Fig. 1F). Rv1324m showed a single band that was consistent with the molecular weight of Rv1324. Moreover, the antioxidant activities of Rv1324m and Rv1324 were assessed via an Fe^3+^ reducing power assay. Rv1324 displayed specific activity toward the increase in Fe^2+^ (Fig. 1H). However, Rv1324m did not exhibit increased activity with increasing amounts of the protein (Fig. 1H). These results confirm that the antioxidant active site of Rv1324 was Cys_78_.

### The effect of Rv1324 on the growth of mycobacteria *in vitro*.

**FIG 2:**
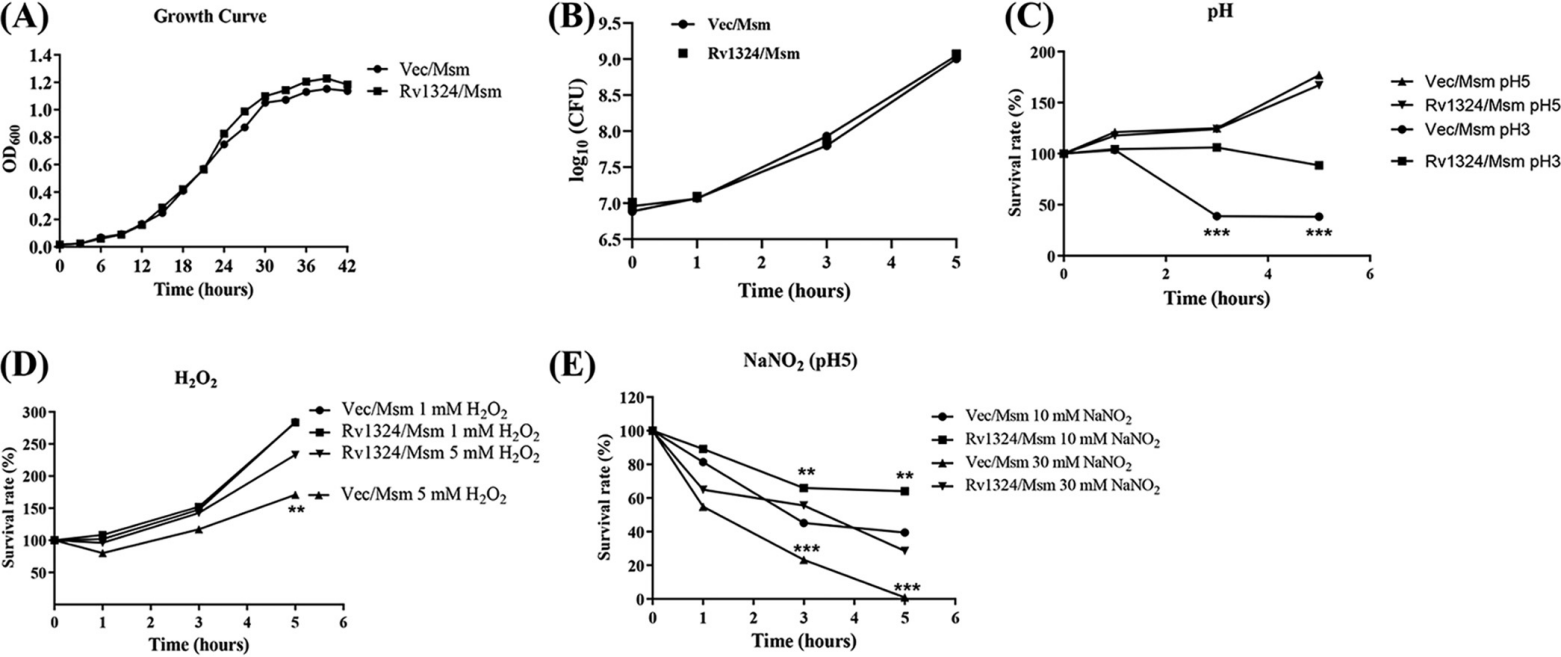
Growth of recombinant M. smegmatis under normal and stressed conditions. (A) The growth of Rv1324/Msm and Vec/Msm at 37°C in 7H9 broth was monitored by determining the OD_600_ at intervals of 3 h until saturated. (B) The growth of Rv1324/Msm and Vec/Msm in 7H9 media. The bacteria were harvested and suspended in 7H9 media to an OD_600_ of 0.5 for continuous growth. After 1, 3, and 5 h of incubation, a volume of 0.1 mL of the culture was removed for CFU counting. (C) The survival of Rv1324/Msm and Vec/Msm at low pH conditions. The bacterial cultures were harvested and suspended in 7H9 media (pH 3.0 or pH 5.0) to an OD_600_ of 0.5 for continuous growth. After 1, 3, and 5 h of incubation, a volume of 0.1 mL of the culture was removed for CFU counting. (D) Survival of Rv1324/Msm and Vec/Msm after exposure to hydrogen peroxide. Aliquots (30 mL) of cultures at an OD_600_ of 0.5 were exposed to 1 or 5 mM hydrogen peroxide (H_2_O_2_). After 1, 3, and 5 h of incubation, a volume of 0.1 mL of the culture was removed for CFU counting. (E) Survival of Rv1324/Msm and Vec/Msm after exposure to sodium nitrite. Aliquots (30 mL) of cultures at an OD_600_ of 0.5 were exposed to 10 and 30 mM acidified (pH 5) sodium nitrite (NaNO_2_). After 1, 3, and 5 h of incubation, a volume of 0.1 mL of the culture was removed for CFU counting. The differences between the groups at each time point were calculated via a two-way ANOVA. Asterisks denote the statistically significant differences between the Vec/Msn and Rv1324/Msm, under pH 3.0 conditions, that were exposed to 5 mM H_2_O_2_ and 10 or 30 mM NaNO_2_. **, *P* < 0.01; ***, *P* < 0.001.

Since the overexpression of the Rv1324 protein in the M. smegmatis strain might influence bacterial growth, the growth of Rv1324/Msm *in vitro* was determined. As shown in Fig. 2A and in Fig. 2B, the growth curve and bacterial count of the Rv1324/Msm strain were similar to those of the Vec/Msm strain, revealing that the overexpression of Rv1324 on M. smegmatis did not affect bacterial growth *in vitro*.

Within the phagosome, the invading microbe is exposed to a hostile environment, including reactive oxygen species, reactive nitrogen species, and low pH conditions. To further investigate the effect of the Rv1324 protein in the M. smegmatis strain on survival in host cells, we analyzed growth characteristics between the Rv1324/Msm and Vec/Msm strains under those stress conditions. The survival ability of the Rv1324/Msm and Vec/Msm strains growing in 7H9 media at a pH of 3.0 and 5.0 was assessed at three time points during a 5 h period of incubation. Differences in the growth of the two strains in 7H9 media at a pH of 5.0 were not observed at any time (Fig. 2C). However, the survival rate of Rv1324/Msm was significantly higher than that of Vec/Msm in 7H9 media at a pH of 3.0 at 3 h and 5 h (Fig. 2C). Similarly, the susceptibilities of the two strains to ROS and RNS were also observed following 5 h of exposure to 1 mM and 5 mM H_2_O_2_ or 10 mM and 30 mM NaNO_2_ (pH 5), respectively. Compared to Vec/Msm, the survival of Rv1324/Msm was strongly enhanced in 7H9 media exposed to 5 mM H_2_O_2_ or to 10 mM or 30 mM NaNO_2_ (Fig. 2D and E). Taken together, these results indicate that the overexpression of Rv1324 in M. smegmatis could contribute to an increased resistance to any of the tested stresses.

### The role of Rv1324 in mycobacterial persistence in lung tissue.

**FIG 3:**
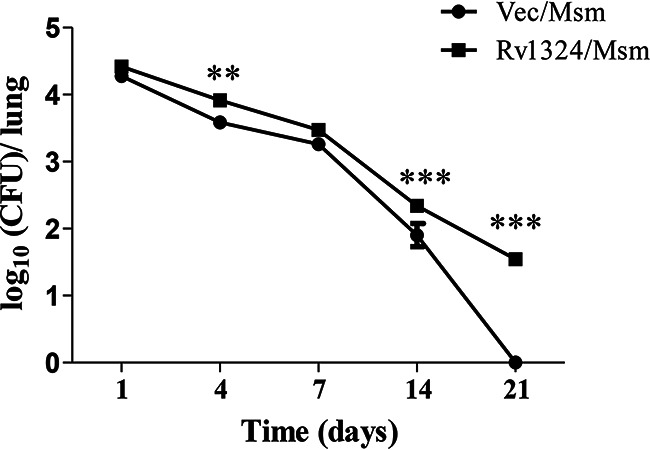
Presence of recombinant M. smegmatis persistence in mouse lungs. C57BL/6J mice were challenged with recombinant Ves/Msm or Rv1324/Msm with a dose of 5 × 10^9^ CFU/day/mouse for 4 days. Mice challenged with PBS were used as a negative control. The mice from each group were sacrificed, and the whole right lobes of their lungs were obtained sterilely and were homogenized in PBS buffer. The bacterial loads in the lung tissue were determined on days 1, 4, 7, 14, and 21 postinfection (3 mice/group/time point). The error bars show the SEM of three independent experiments with three mice per group. The differences between the groups at each time point were calculated via a two-way ANOVA. Asterisks denote the statistically significant differences between the mice infected with Vec/Msn and those infected with Rv1324/Msm. **, *P* < 0.01; ***, *P* < 0.001.

To determine the roles of Rv1324 in the persistence of M. smegmatis in lung tissue, the mice in the PBS group, Vec/Msm group, and Rv1324/Msm group were infected with PBS, a high dose of Vec/Msm, or a high dose of Rv1324/Msm for 4 days in an aerosolized model. The bacterial loads in the lung tissue were assessed on days 1, 4, 7, 14, and 21 after infection (Fig. 3). The results showed that there was more Rv1324/Msm persistence than Vec/Msm persistence in the lungs of the mice. Moreover, on days 4, 14, and 21 postinfection, the bacterial loads in the mice challenged with Rv1324/Msm were significantly increased, compared with those in the mice infected with Vec/Msm. As opposed to the almost complete clearance of bacteria in the lungs of the mice infected by Vec/Msm on day 21, the bacterial loads were sustained in the lungs of the mice that were administered Rv1324/Msm on day 21. This implies that the Rv1324 protein benefited the persistence of M. smegmatis in the lung tissues of the mice.

### The roles of Rv1324 in lung injury and inflammation.

**FIG 4:**
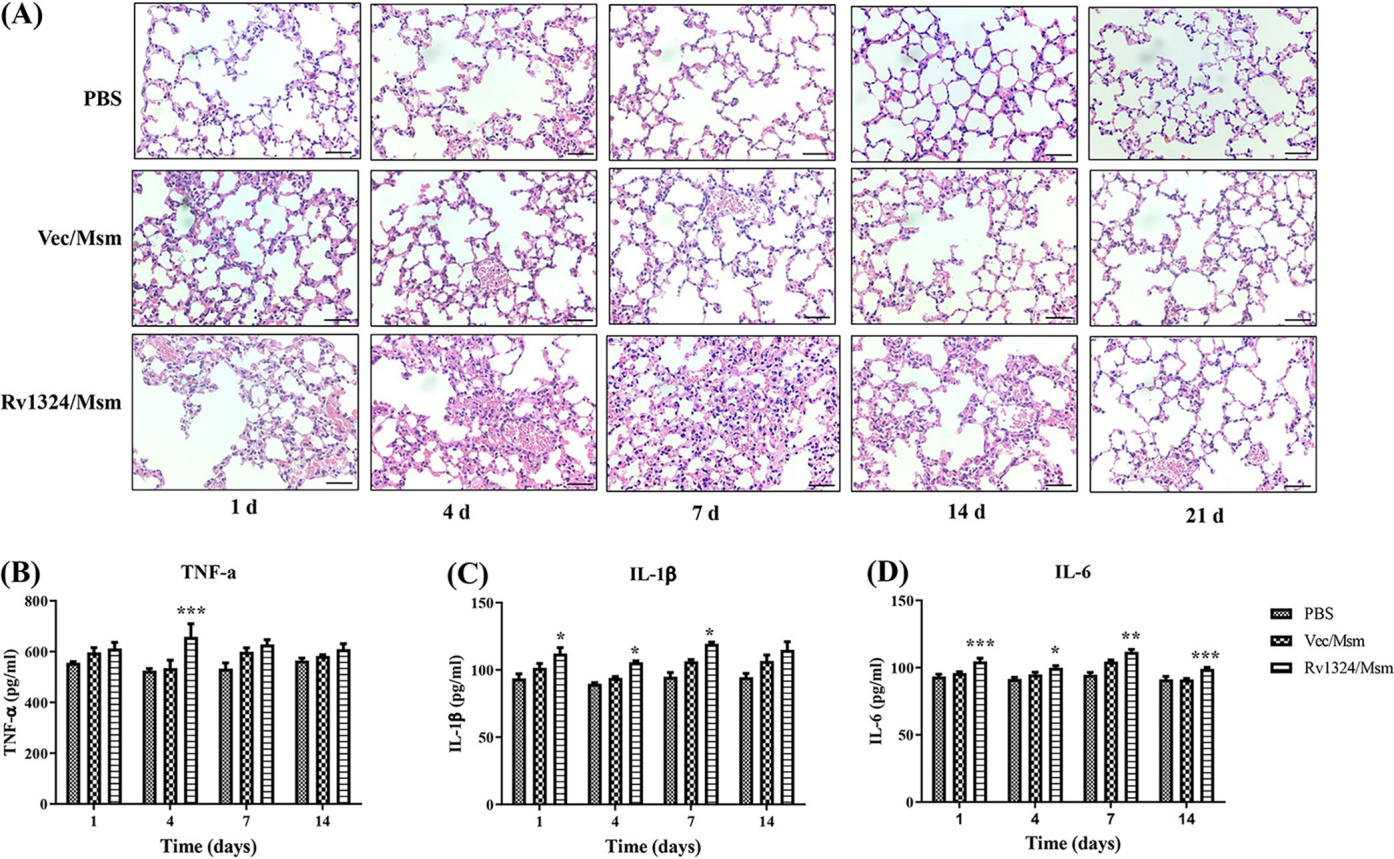
Lung histology and inflammatory responses of recombinant M. smegmatis-infected mice. Each C57BL/6J mouse was challenged with PBS, recombinant Ves/Msm, or Rv1324/Msm with a dose of 5 × 10^9^ CFU per day for 4 days. One left lobe of lung was obtained and fixed with 4% PFA for pathological analysis. Moreover, serum was collected for the quantification of inflammatory indicators. (A) The lung tissues of mice infected with Ves/Msm or Rv1324/Msm were collected on days 1, 4, 7, 14, and 21 postinfection and stained with H&E. The tissue slides were observed using 40 × microscope objectives. Scale bar = 100 μm. (B–D) Quantification of the inflammatory indicators TNF-α (B), IL-1β (C), and IL-6 (D) in mice infected with recombinant Ves/Msm or Rv1324/Msm. Serum was collected from the infected mice (*n* = 3 per group), and the cytokines TNF-α, IL-1β, and IL-6 were determined. The error bars show the SEM of three independent experiments with three mice per group. The differences between the groups at each time point were calculated via a two-way ANOVA. Asterisks denote the statistically significant differences between the mice infected with Vec/Msn and those infected with Rv1324/Msm.*, *P* < 0.05; **, *P* < 0.01; ***, *P* < 0.001.

The lung histopathology was observed at different time points postinfection. The results showed that the lungs of the mice challenged with high doses of both Rv1324/Msm and Vec/Msm had an inflammatory response with evident angiectasis, hyperemia, and alveolar septum enlargement after continuous infection for 4 days, compared to the PBS control group (Fig. 4A). However, an acute inflammatory response with cellular edema and massive infiltration and hemorrhage was observed in Rv1324/Msm-infected mice on day 4 after the challenge (Fig. 4A). In contrast, an inflammatory response with slight infiltration and hemorrhage was shown in the lungs of mice infected with Vec/Msm on day 4 (Fig. 4A). On day 14 after the challenge, the inflammation disappeared in the Vec/Msm-infected mice, but there was still inflammation in the Rv1324/Msm-infected mice (Fig. 4A). This finding indicates that the mice challenged with Rv1324/Msm exhibited more serious pathological and histopathological lesions than did the mice challenged with Vec/Msm.

To quantify the degree of inflammation, the levels of the cytokines IL-6, TNF-α, and IL-1β were measured in the sera of mice on days 1, 4, 7, and 14 after infection. The levels of the proinflammatory cytokines IL-6, TNF-α, and IL-1β in the mice challenged with Rv1324/Msm were significantly higher (*P* < 0.05) than those observed in the control mice (PBS and Vec/Msm) (Fig. 4B–D). These results reveal that Rv1324/Msm activated the proinflammatory response.

### The roles of Rv1324 on ROS generation and oxidative stress.

**FIG 5:**
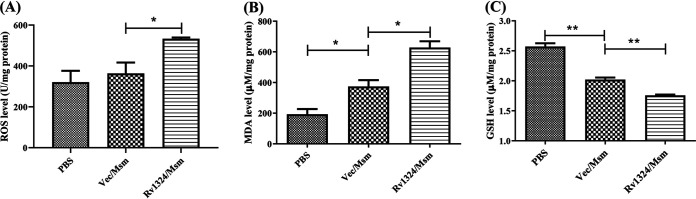
The generation of reactive oxygen species (ROS) and the oxidative stress in the lungs of mice infected with recombinant M. smegmatis. C57BL/6J mice were challenged with PBS, recombinant Ves/Msm, or Rv1324/Msm with a dose of 5 × 10^9^ CFU/day/mouse for 4 days. The mice from each group were sacrificed, and whole right lobes of lungs were obtained sterilely and homogenized in PBS buffer. (A) The ROS in the lung homogenates of mice were measured using a Mouse ROS ELISA Kit (*n* = 3 per group). (B) The production of MDA was detected using an MDA Assay Kit (*n* = 3 per group). (C) The GSH levels were detected using a GSH Assay Kit (*n* = 3 per group). The error bars show the SEM of three independent experiments with three mice per group. The differences between the groups at each time point were calculated via a two-way ANOVA. *, *P* < 0.05; **, *P* < 0.01.

The level of ROS was determined in lung homogenates of mice challenged with PBS, Vec/Msm, or Rv1324/Msm. In the Rv1324/Msm-infected mice, the levels of ROS in the lung homogenates were significantly greater (*P* < 0.05) than those observed in the PBS-treated mice and in the Vec/Msm-infected mice (Fig. 5A). As a marker of oxidative stress, MDA was enhanced (*P* < 0.05) in the lungs of the mice challenged with Rv1324/Msm (Fig. 5B). GSH is an antioxidant compound, and its level was decreased (*P* < 0.05) in the Rv1324/Msm-infected mice (Fig. 5C). These data indicate that Rv1324/Msm could induce ROS generation and oxidative stress in mice.

### The role of Rv1324 in ferroptosis in lung tissue.

**FIG 6:**
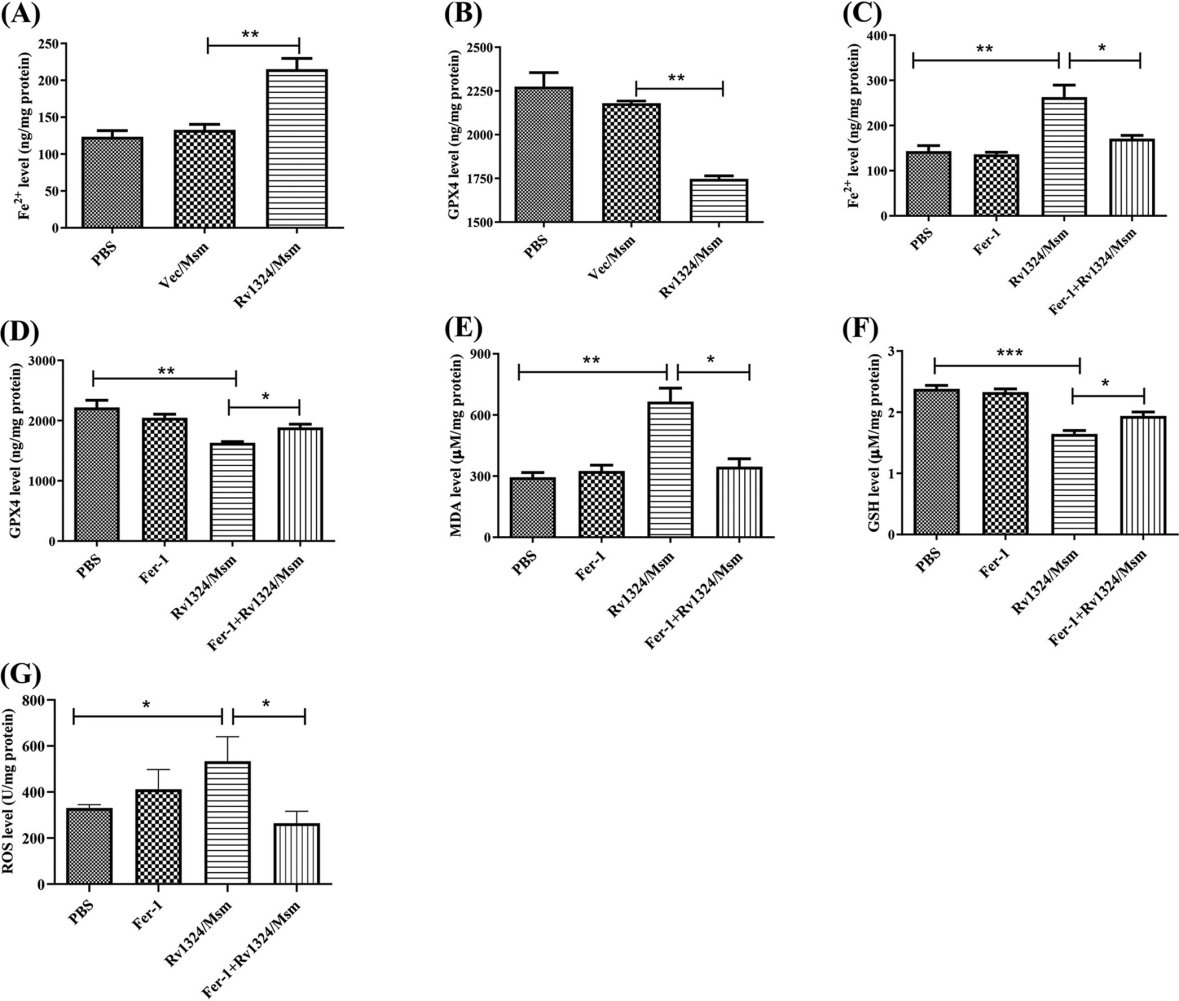
Ferroptosis in the lungs of mice infected with recombinant M. smegmatis. Each C57BL/6J mouse was challenged with PBS, recombinant Ves/Msm, or Rv1324/Msm with a dose of 5 × 10^9^ CFU per day for 4 days. The mice from each group were sacrificed, and whole right lobes of lungs were obtained sterilely and homogenized in PBS buffer. The ferroptosis levels of lungs were evaluated by detecting the biomarkers of ferroptosis. (A) The Fe^2+^ levels of the lungs were determined using an Iron Assay Kit. (B) The glutathione peroxidase 4 (GPX4) activities of the lungs were measured using a GPX4 ELISA Kit. (C–G) The levels of Fe^2+^ (C), MDA (D), GSH (E), GPX4 (F), and ROS (G) in the lungs of Rv1324/Msm-infected mice treated with or without Fer-1 were analyzed using the corresponding kits. The error bars show the SEM of three independent experiments with three mice per group. The differences between the groups at each time point were calculated via a two-way ANOVA. *, *P* < 0.05; **, *P* < 0.01; ***, *P* < 0.001.

Ferroptosis is characterized by an iron-dependent increase in oxidative stress and lipid peroxidation (8–10). To confirm the effect of Rv1324/Msm on ferroptosis, we measured the levels of ferroptosis biomarkers, including the iron content and GPX4 levels, in lung tissues, which are considered to be the signs and main regulators of ferroptosis (8–10). The Fe^2+^ levels significantly increased (*P* < 0.05), and the GPX4 activity significantly decreased (*P* < 0.05) in the mice challenged with Rv1324/Msm, compared to the PBS and Vec/Msm groups (Fig. 6A and B). Therefore, these results suggest that Rv1324/Msm promoted ferroptosis in the lung tissues of mice.

Subsequently, the mice were pretreated with Fer-1, an inhibitor of ferroptosis, and they were then challenged with Rv1324/Msm for 4 days to further investigate the effect of Rv1324/Msm on ferroptosis in mice. The Fer-1 treatment inhibited the Fe^2+^ overload, inhibited the accumulation of MDA and ROS, and increased the activity of GSH and GPX4 in the Rv1324/Msm-infected mice (Fig. 6C–G). These results further suggest that Rv1324/Msm induced ferroptosis in the lungs of mice.

### Fer-1 attenuated, Rv1324-induced lung injury, inflammation, and mycobacterial persistence in lung tissue.

**FIG 7:**
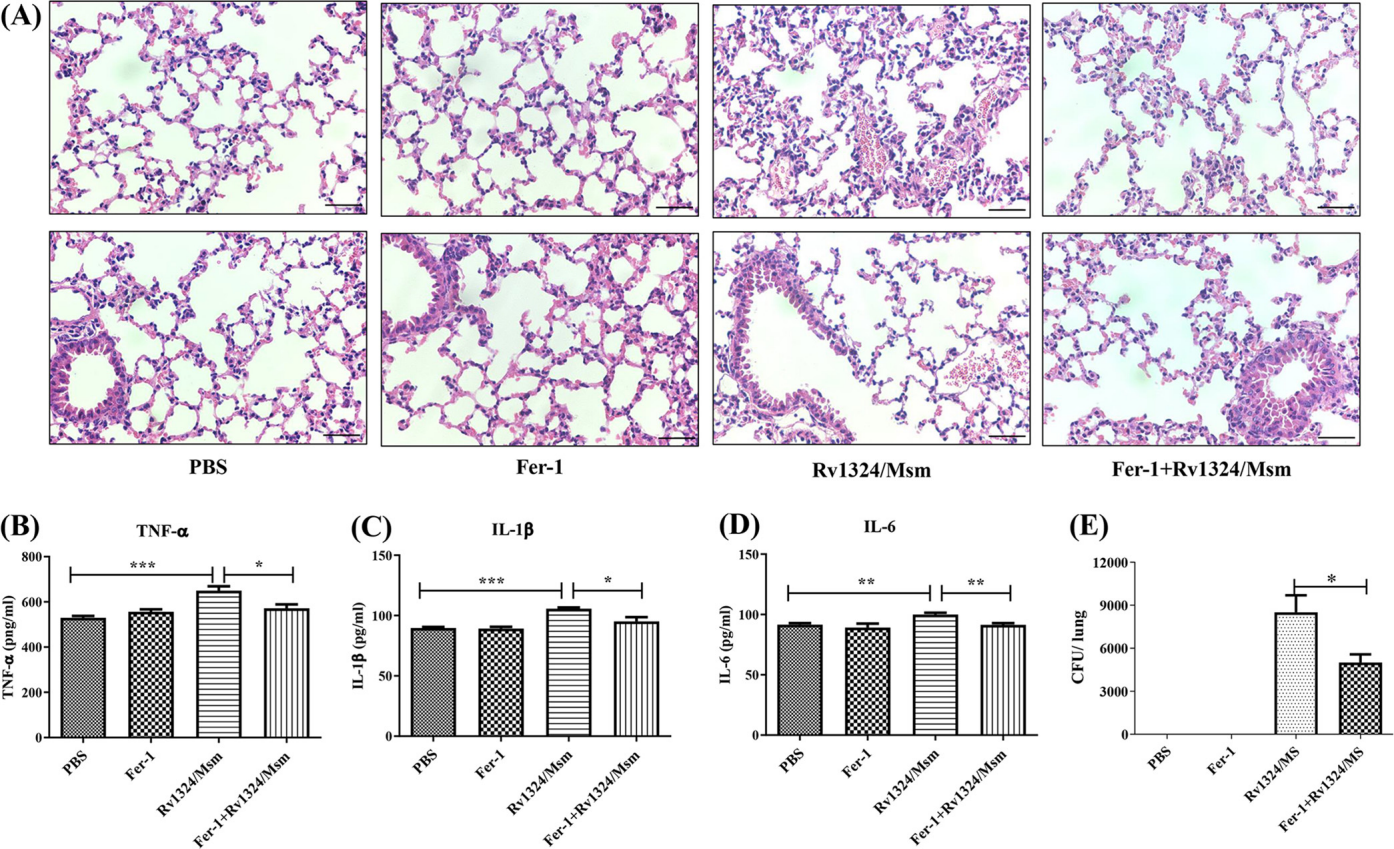
The effect of Fer-1 on lung histology, inflammatory responses, and bacterial loads in the lungs of mice challenged with Rv1324/Msm. The mice were administered with Fer-1 (an inhibitor of ferroptosis, 3 mg/kg/mouse per day) or PBS via intravenous injection for 3 consecutive days. Then, the Fer-1-treated mice and PBS-treated mice were divided into two groups and were infected with either PBS or Rv1324/Msm with a dose of 5 × 10^9^ CFU per day for 4 days. On day 4 postinfection, the mice in the PBS group, Fer-1group, Rv1324/Msm group, and Fer-1+ Rv1324/Msm group were sacrificed. Then, lung tissues and sera were collected. (A) Lung tissues of Rv1324/Msm-infected mice treated with or without Fer-1 were collected on day 4 postinfection and were stained with H&E. The tissue slides were observed using 40 × microscope objectives. Scale bar = 100 μm. (B–D) Quantification of inflammatory indicators in Rv1324/Msm-infected mice treated with or without Fer-1. The sera of mice (*n* = 3 per group) were collected, and the cytokines TNF-α (B), IL-1β (C), and IL-6 (D) were determined. The error bars show the SEM of three independent experiments with three mice per group. The differences between the groups at each time point were calculated via a two-way ANOVA. *, *P* < 0.05; **, *P* < 0.01; ***, *P* < 0.001. (E) The bacterial loads in the lung tissues of mice from the PBS group, Fer-1 group, Rv1324/Msm group, and Fer-1+Rv1324/Msm group were determined on day 4 after infection (3 mice/group/time point). The error bars show the SEM of three independent experiments with three mice per group. The differences between the groups at each time point were calculated via *t* tests. *, *P* < 0.05.

Increasing evidence has shown that ferroptosis plays an important role in inflammation (30). We observed lung damage and assessed lung inflammation and the content of proinflammatory factors in the sera of mice pretreated with Fer-1. On day 4 postinfection, the lung hemorrhage and infiltration of inflammatory cells into the lungs of Rv1324/Msm-infected mice pretreated with Fer-1 were reduced, compared with those in the mice infected with Rv1324/Msm alone (Fig. 7A). In addition, the Fer-1 treatment significantly reduced (*P* < 0.05) the levels of IL-6, IL-1β, and TNF-α in the sera of mice challenged with Rv1324/Msm (Fig. 7B–D). These data demonstrate that Rv1324/Msm induced lung damage and an inflammatory response by activating ferroptosis.

Next, the effect of ferroptosis induced by Rv1324/Msm on mycobacterial persistence in the lung tissues was also determined. As shown in Fig. 7E, the Fer-1 treatment reduced the bacterial loads in the lungs of the mice challenged with Rv1324/Msm. This finding suggests that the persistence of Rv1324/Msm in the lung tissues of the mice was mediated by the induction of ferroptosis.

## DISCUSSION

In this study, we cloned, purified, and characterized the Mtb Rv1324 protein with antioxidant activity. An alignment analysis revealed that the amino acid sequence of the Rv1324 protein shared 24.6% identity and 61.1% similarity with TrxA as well as 27.6% identity and 61.0% similarity with TrxC. However, the canonical Cys-X-X-Cys (CXXC) active site of Trxs was replaced by a Ser-X-X-Cys (SXXC) sequence in Rv1324. Although CXXC is the major redox motif that was used for the formation, reduction, and isomerization of the disulfide bonds, CXXC-derived motifs, such as CXXS, SXXC, CXXT, and TXXC, have been found in several redox proteins, including glutaredoxins, arsenate reductases, methionine sulfoxide reductases (CXXS), peroxiredoxins (SXXC and TXXC), glutathione peroxidases (CXXT) (31), and several uncharacterized thioredoxin-like proteins. Our results showed that Rv1324 has one redox-active cysteine. Thus, it was unable to stimulate insulin reduction in the presence of dithiothreitol, which was accomplished by the two cysteines of the CXXC active site. However, it had antioxidant activity that was important for protecting Mtb against oxidative stress. To further confirm the antioxidant activity site of Rv1324, we constructed an E. coli BL21(DE3) strain overexpressing the Rv1324 mutant protein (Rv1324m). In Rv1324m, the Cys_78_ in the Rv1324 protein was replaced by Ala_78_, using a site-directed mutagenesis strategy. Moreover, we did not detect antioxidant activity in Rv1324m, indicating that the antioxidant activity site of the Rv1324 protein was Cys_78_. In addition, we generated a recombinant Rv1324/Msm strain to overexpress the Rv1324 protein in M. smegmatis. The Rv1324 protein was found to be located in the cell envelope of the Rv1324/Msm strain, which was consistent with the results of a previous report (24).

During macrophage phagocytosis, mycobacteria are exposed to acidic conditions (32). In this study, to investigate the effect of Rv1324 on mycobacterial survival in the host, the survival of the Rv1324/Msm strain under low pH (pH 3.0 and pH 5.0) conditions was determined. The results showed that the survival rates of the two strains were increased in 7H9 media at a pH of 5.0 and decreased in 7H9 media at a pH of 3.0, as M. smegmatis can be grown over a wide pH range, but its growth is inhibited at pH values below 4.5 (33, 34). In addition, we found that there were no obvious differences in the growth of the two strains in the 7H9 media at a pH of 5.0 at any time point. However, the survival of Rv1324/Msm was significantly higher than that of Vec/Msm in the 7H9 media at a pH of 3.0 at 3 h and 5 h. These results indicated that the Mtb Rv1324 protein played a role in mycobacterial survival in acidic environments. After macrophage engulfment, Mtb is exposed not only to an acidic environment but also to antimicrobial redox stresses, including reactive oxygen species (ROS) and reactive nitrogen species (RNS). ROS, hydrogen peroxide (H_2_O_2_), superoxide (O^2−^), and hydroxyl radicals (OH) are mainly produced by NADPH oxidase (the main generator of ROS inside host cells). RNS, specifically nitric oxide (NO), is mainly generated by inducible nitric oxide synthase (iNOS) (35). Several of the redox proteins of Mtb are responsible for detoxifying the host redox stresses and enhancing the survival of Mtb in the host cells (36). In this study, we found that the Rv1324 protein had antioxidant activity and that Rv1324 increased the survival of M. smegmatis that was exposed to exogenously provided H_2_O_2_ and nitric oxide, demonstrating that the Rv1324 protein is a molecule that is involved in the mycobacterial antioxidant defense and might benefit Mtb against ROS and RNS in the host.

The molecules of the antioxidant defense systems of microorganisms were originally considered an important conserved family for protection against ROS or RNS by reducing peroxides to harmless products (17, 22, 29), but little is known about the specific roles of these molecules in bacterial virulence during infection. To further confirm the effect of the Rv1324 protein on the survival and dissemination of mycobacteria in the host, we constructed a model of C57BL/6J mice infected with Rv1324/Msm to investigate the function of Rv1324 during infection. The results showed that the Rv1324 protein enhanced the survival and promoted the colonization of M. smegmatis in the lung but also induced lung lesions and proinflammatory responses in mice. These results indicated that Rv1324 was adequate to confer pathogenic characteristics to M. smegmatis and could be a virulence factor of Mtb. In addition, our results showed that the overexpression of Rv1324 in M. smegmatis also induced ROS accumulation and oxidative stress in the lungs of mice, which further indicated the role of Rv1324 as a mycobacterial virulence factor. It is well-known that ROS represent a double-edged sword. Although ROS can kill infectious agents within host cells (37), they also cause host damage. During an infection, high levels of ROS are produced to counteract and kill the mycobacteria (38). If the ROS levels are overwhelmed by the mycobacteria antioxidant systems, then the pathogen can continue to survive and proliferate in the host (39). However, excess ROS induce an overactivated immune response (40), leading to host cell damage or death (39). Recently, a report showed a similar bactericidal response in which a thioredoxin-like protein from Edwardsiella piscicida was found to act as a virulence factor to interfere with host antibacterial signaling, which could inhibit cellular redox signaling and thereby lead to increased ROS accumulation in infected host cells (41). This implies that bacterial Trx-like proteins not only catalyze protein disulfide reductase but also act as virulence factors during infection. These data imply that the overexpression of Rv1324 in M. smegmatis enhanced the abilities of the mycobacterial antioxidant systems and played a role in mycobacterial survival and dissemination during infection.

During an infection, Mtb-induced host cell death likely plays a crucial role in its pathogenesis. Ferroptosis was identified as one type of programmed necrosis in macrophages in response to Mtb infection (7). It has been confirmed that the association between lung necrosis and Mtb-induced ferroptosis probably contributes to TB pathology and allows Mtb to thrive and spread (6, 7). Compelling evidence indicates that ferroptosis is inextricably linked to oxidative stress (6, 8–10). ROS, as oxidants produced by redox stress, are considered important signals of ferroptosis (8). Iron is necessary for the accumulation of lipid peroxides and for the process of ferroptosis. Excess Fe^2+^ produced in the cell can directly catalyze lipid ROS generation through the Fenton reaction, thereby resulting in the continuous accumulation of intracellular lipid ROS and triggering ferroptosis (42, 43). Interestingly, exogenous iron was necessary for the growth of Mtb (10). Therefore, it was speculated that Mtb-induced macrophage ferroptosis might be triggered by the inhibition of the oxidation of Fe^2+^ by the promotion of excessive ROS. In this study, we found that the Rv1324 protein induced both ROS accumulation and the oxidative stress response. This raised the question of whether the Rv1324 protein benefits mycobacteria to induce the ferroptosis of host cells. To answer this question, we examined the production of biomarkers of ferroptosis, including iron, MDA, GSH and GPX4, in Rv1324/Msm-infected mice. MDA is a final product of lipid peroxidation. GPX4, a phospholipid hydroperoxidase, possesses the unique capability to suppress lipid peroxidation (44). GSH is an antioxidant molecule, and its depletion directly activates lipoxygenases and inhibits GPX4 activity to induce lipid peroxidation (45). It was found that the mice infected with Rv1324/Msm showed significantly higher levels of iron and MDA as well as decreased levels of GSH and GPX4, compared to the mice infected with Vec/Msm. These data indicate that the Rv1324 protein might promote mycobacteria to induce ferroptosis in the lungs of mice.

**FIG 8:**
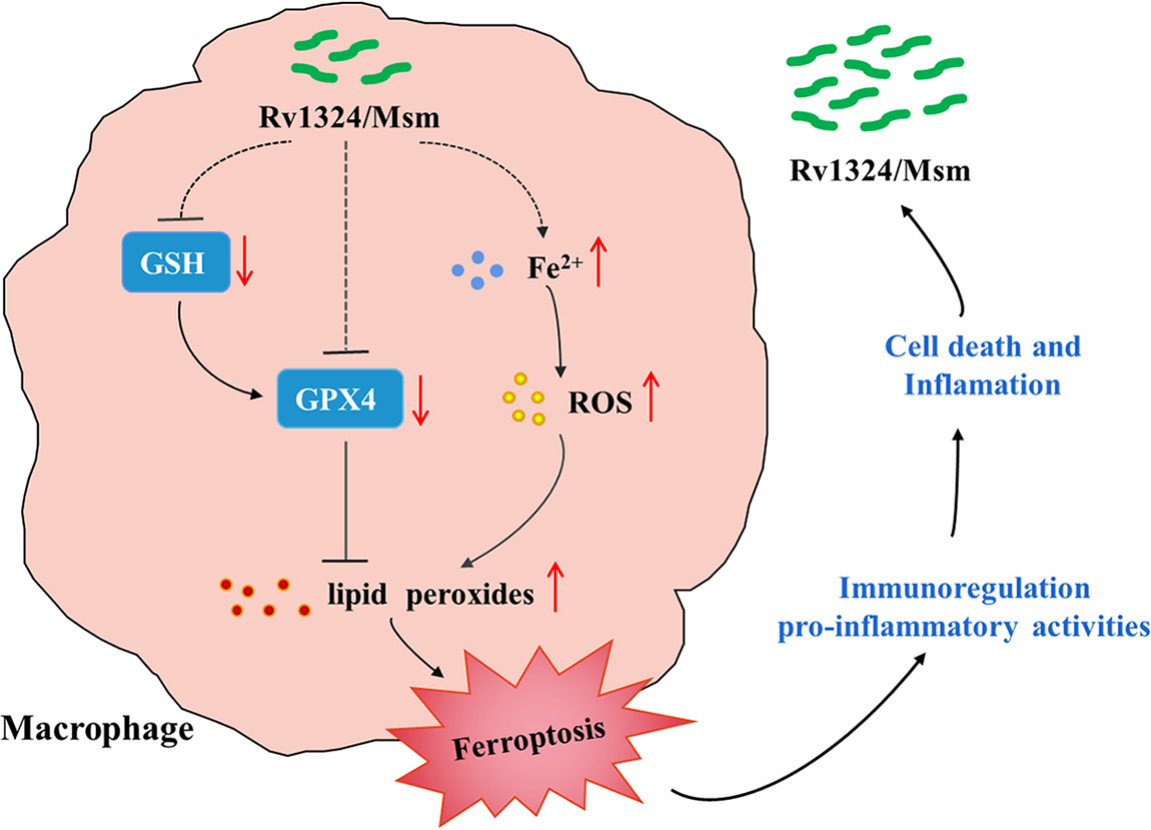
Proposed mechanism for the M. tuberculosis Rv1324 protein. The novel bacterial virulence factor, Rv1324 protein, could induce oxidative stress and increase the ROS production in the lungs of mice infected with Rv1324/Msm, a M. smegmatis strain overexpressing the Rv1324 protein, which might cause the ferroptosis of macrophages, cause proinflammatory responses, lead to pathological lung damage, and lead to the dissemination of the infection in the lungs of mice.

Fer-1 is a well-known ferroptosis inhibitor due to its highly effective suppression of lipid peroxidation (43); therefore, it was utilized to explore the molecular mechanism of ferroptosis. In this study, the mice were treated with Fer-1 before they were challenged with Rv1324/Msm. We found that the Fer-1 treatment inhibited ROS production and the subsequent ferroptosis, which was similar to the results of a previous report (43). Some studies confirm that ferroptosis was also believed to play an important role in inflammation (46–48). Moreover, ferroptosis inhibition suppresses the subsequent immune cell infiltration and the inflammatory response (49–51). We found that Fer-1 treatment attenuated the proinflammatory response, mitigated lung damage, and affected mycobacterial persistence in the lung. Therefore, these data suggest that the Rv1324 protein contributed to mycobacterial persistence and caused a proinflammatory response as well as pathological lung damage in mice by promoting ferroptosis (Fig. 8).

In conclusion, our study demonstrated that the Rv1324 protein has antioxidant activity. The overexpression of Rv1324 in M. smegmatis enhanced the abilities of mycobacterial antioxidant systems and promoted host cell ferroptosis, the inflammatory response, and mycobacterial survival and dissemination during infection. The inhibition of ferroptosis significantly reduced Rv1324/Msm-induced lung injury and the release of inflammatory cytokines in the infected mice. These data indicate that the Rv1324 protein is an important virulence factor involved in mycobacterial survival and dissemination during infection. However, the mechanism by which Rv1324 induces host cell ferroptosis still needs to be investigated. In future studies, we will explore the molecular mechanism by which Rv1324 induces ferroptosis in macrophage cells.

## MATERIALS AND METHODS

### Mice, bacteria, and plasmids.

The specific, pathogen-free (SPF) C57BL/6J mice (6 to 8 weeks old) that were used in this study were purchased from Chang Sheng Biotechnology (Liaoning, China). All of the animal experiments conformed to the NIH Guide for Care and Use of Laboratory Animals and were approved by the Committee on the Ethics of Animal Experiments of Dalian Medical University (permit number: SYXK (Liao) 2018-0007). All of the experiments were conceived to minimize the numbers of animals that were used, and effort was made to minimize both distress and pain for the animals.

The Escherichia coli (E. coli) strains NovaBlue and BL21(DE3) were obtained from Novagen (Malaysia) and grown in LB broth or on LB agar plates. Wild-type M. smegmatis mc^2^155 was obtained from ATCC and grown in 7H9 broth containing 0.05% Tween 80 or on 7H11 agar plates. Antibiotics were purchased from Sigma and used at the following final concentrations: ampicillin (Amp), 100 μg/mL; kanamycin (Kan), 50 μg/mL for NovaBlue and 25 μg/mL for M. smegmatis mc^2^155. The plasmids pJET1.2/blunt and pColdII were from Thermo Fisher Scientific and TaKaRa, respectively. The plasmid pVV2 carrying an hsp60 promoter for the expression of a target protein in the mycobacteria was obtained from Colorado State University, USA.

### Construction of recombinant Rv1324/Msm strains.

The Rv1324 gene was PCR-amplified using M. tuberculosis H37Rv genomic DNA as the template with the specific forward primer 5′-TTTCATATGGTGACGCGTCCGCGACCC-3′ and the reverse primer 5′-AGAAGCTTTCAGTACAGCGCGTTGGCGAG-3′ (The underlining showed the specific sites recognized by restriction enzymes NdeI and HindIII. NdeI recognition sites is CATATG. HindIII recognition sites is AAGCTT). The purified Rv1324 PCR product was then ligated with the cloning vector pJET1.2/blunt (TaKaRa, Japan) and transformed into NovaBlue (TaKaRa, Japan). After the confirmation of the Rv1324 sequence in the pJET-Rv1324 plasmid via sequencing, the plasmid was digested by NdeI (TaKaRa, Japan)and HindIII (TaKaRa, Japan), and the Rv1324 was subcloned into the NdeI and HindIII sites of the pVV2 vector, a M. smegmatis-E. coli shuttle vector, yielding the expression vector pVV2-Rv1324. The pVV2-Rv1324 and empty vector pVV2 were transformed into M. smegmatis mc^2^ 155 cells via electroporation at 2500 V, 1000 Ω, and 25 microfarads using a 2 mm electroporation cuvette (Bio-Rad), thereby generating the recombinant M. smegmatis strains Rv1324/Msm and Vec/Msm, respectively.

The expression of Rv1324 in the recombinant Rv1324/Msm strain was assessed via SDS-PAGE and Western blotting analyses. The samples were prepared as follows. After the Rv1324/Msm cells were harvested, the cells were sonicated in lysis buffer and then centrifuged at 15,000 × *g* for 20 min. The supernatant containing the Rv1324 protein was collected.

The concentration of whole-cell lysate proteins was determined using a BCA Protein Assay Kit (Vazyme, China), using bovine serum albumin (BSA) as a standard. The assay was performed in 96-well plates. 200 μL of a dye solution was added to 25 μL of the protein sample. The mixture was incubated at 37°C for 30 min, after which the absorbance of the solutions at 562 nm was evaluated with a microplate spectrophotometer (MultiskanFC, Thermo Scientific).

Whole-cell lysate proteins (20 μg) were added to sodium dodecyl sulfate-polyacrylamide gel electrophoresis (SDS-PAGE) gels to analyze the expression of the Rv1324 protein. The whole-cell lysate proteins of Vec/Msm were used as control proteins. For the SDS-PAGE analysis, the gel with the whole-cell lysate proteins of the Vec/Msm or Rv1324/Msm strains was stained with Coomassie blue R-250. For the Western blotting, the proteins in the SDS-PAGE gels were electrotransferred to nitrocellulose membranes. Then, the membrane was incubated with the monoclonal anti-polyhistidine antibody clone HIS-1. This was followed by incubation with an alkaline phosphatase-conjugated anti-mouse IgG antibody. Finally, the Rv1324 protein was visualized in a NBT/BCIP solution.

### Localization of the Rv1324 protein in Rv1324/Msm.

The bacterial cells of the Rv1324/Msm strain were inoculated into 200 mL of 7H9 broth and grown with shaking at 37°C until the OD_600_ reached 0.5. The cells were collected and sonicated in lysis buffer at 4°C. Subsequently, the subcellular fractions were harvested via centrifugation.

The preparation of the subcellular fractions from the recombinant Rv1324/Msm was performed as previously described (52), with modification. Briefly, the cell lysates of Rv1324/Msm were centrifuged at 3,000 × *g* for 10 min at 4°C to collect the supernatant as whole-cell lysate (WCL). The WCL was then centrifuged at 27,000 × *g* for 1 h at 4°C to obtain the cell wall pellet (CW), and the soluble (SOL) fraction was then centrifuged at 100,000 × *g* for 2 h at 4°C to obtain the cell membrane (CM) fraction. The CW and CM fractions were washed once with lysis buffer, recentrifuged, and subsequently resuspended in 0.5 mL of lysis buffer. The concentrations of proteins in the fractions were quantified using a BCA Protein Assay Kit (Vazyme, China), using bovine serum albumin (BSA) as the standard. The fractions were assessed via Western blotting. Finally, the protein bands were visualized using WesternBright ECL detection reagents (Advansta, China).

### *In vitro* growth and stress assays of the Rv1324/Msm strain.

To assess the growth of Rv1324/Msm *in vitro*, it was inoculated in 7H9 broth containing Kan with an initial optical density at 600 nm (OD_600_) of approximately 0.02 and grown with shaking at 37°C for 36 h. The culture was monitored by determining the OD_600_ value at various time points (every 3 h). The Vec/Msm strain, which was used as a control, was grown and assessed under the same conditions as was Rv1324/Msm.

For the stress assays, Rv1324/Msm cells were harvested at an OD_600_ of 0.5 and then washed with 7H9 under different physiological stress conditions, including media with the pH adjusted to 5 or 3 (26, 53), supplemented with 1 or 5 mM H_2_O_2_ for oxidative stress (54), and supplemented with 10 or 30 mM sodium nitrite (acidified sodium nitrite at pH 5) for nitrosative stress (54). The bacterial cells were resuspended to an OD_600_ of 0.5 in 30 mL 7H9 broth under these different physiological stress conditions. The cultures were then grown at 37°C, and 1 mL samples were removed at 0, 1, 3, and 5 h to count the CFU on the 7H11 agar plates. The Vec/Msm strain, which was used as a control, was grown and assessed under the same conditions.

### Expression, purification, and detection of the recombinant Rv1324 protein.

The Rv1324 gene in pVV2-Rv1324 was subcloned into the pColdII expression vector to generate pCold-Rv1324. E. coli BL21(DE3) with pCold-Rv1324 was induced by 0.5 mM IPTG at 16°C to produce the Rv1324 protein. After harvesting, the cells were sonicated in lysis buffer and then centrifuged at 15,000 × *g* for 20 min. The supernatant containing the Rv1324 protein was collected for the purification of Rv1324.

According to the manufacturer’s instructions, the supernatant was applied to a column containing 1 mL Ni-NTA SuperFlow resin. The column was washed twice with 10 volumes of washing buffer (lysis buffer with 45 mM imidazole). Then, the Rv1324 protein was eluted with 8 volumes of eluting buffer (lysis buffer with 200 mM imidazole).

The purified Rv1324 protein was assessed via SDS-PAGE and Western blotting. For the SDS-PAGE analysis, the gel with the purified Rv1324 protein was stained with Coomassie blue R-250. For the Western blotting, the purified Rv1324 protein in the SDS-PAGE gel was electrotransferred to a nitrocellulose membrane. Then, the membrane was incubated with the monoclonal anti-polyhistidine antibody clone HIS-1, and this was followed by incubation with the alkaline phosphatase-conjugated anti-mouse IgG antibody. Finally, the Rv1324 protein was visualized in a NBT/BCIP solution. The purified Rv1324 protein was also identified via matrix-assisted laser desorption/ionization time-of-flight mass spectrometry (MALDI-TOF MS), which was performed by Shanghai Applied Protein Technology Co., Ltd.

The concentration of the purified Rv1324 protein was quantified using a BCA Protein Assay Kit (Vazyme, China).

### Determination of Rv1324 protein activity.

The total antioxidant capacity was estimated via a FRAP (ferric reducing ability of plasma) assay. It is based on the use of antioxidant substances to reduce ferric-tripyridyltriazine (Fe^3+^-TPTZ) and produce Fe^2+^-TPTZ under acidic conditions. Fe^2+^-TPTZ is a blue-violet complex, and it is detected by reading the absorbance at 593 nm. In this study, the antioxidant activity of Rv1324 was measured with an Fe^3+^ reducing, power-based Total Antioxidant Capacity Detection Kit (Nanjing Jiancheng Bioengineering Co., Ltd.), according to the manufacturer’s protocol. In brief, 20 μL recombinant Rv1324 protein at different concentrations (0.5, 1, 1.5, 2, 2.5, and 3 mg/mL) was added to a 200 μL FRAP solution (Fe^3+^-TPTZ diluent). After incubation at 37°C for 10 min, the absorbance at 593 nm was detected by a microplate spectrophotometer (MultiskanFC, Thermo Scientific). In the FRAP assay, the total antioxidant capacity is represented by the concentration of FeSO_4_. Therefore, the OD_593_ value of FeSO_4_ at different concentrations (0.15, 0.3, 0.6, 0.9, 1.2, and 1.5 mM) was measured. In addition, the standard curve of the FeSO_4_ concentration-OD_593_ was obtained and used to calculate the antioxidant activity of the Rv1324 protein. As a negative control, the assay was performed without the Rv1324 protein. The assay was conducted in triplicate, and the data are presented as the means ± SEMs.

### Confirmation of the Rv1324 protein active site.

The Rv1324 mutant (Rv1324m) at the 78th residue was generated by substituting the cysteine at position 78 of Rv1324 with alanine. The forward primer (primer 1, 5′-ACCCCGCAGCGAGGTAGCCGTCGACTTGCTTGA-3′) and the reverse primer (primer 2, 5′-GACCACAGCAACACCACCACCGGCACTTCGT-3′) were designed by replacing codon TGC (for Cys_78_) to GCC (for Ala_78_) (The underlining showed the mutant sites of Rv1324 gene). The pJET-Rv1324 plasmid was used as the template to carry out site-directed mutagenesis, using a TaKaRa MutanBEST Kit (TaKaRa, Japan). The pJET-Rv1324m was amplified via PCR with an initial 5 min incubation at 94°C. This was followed by 30 cycles of 94°C for 30 s, 64°C for 30 s, and 72°C for 5 min, with a final step of 72°C for 5 min. After blunting, kinating, and ligating, the PCR product was transformed into NovaBlue competent cells. The cells were plated on LB agar containing Amp (100 μg/mL) and incubated at 37°C overnight. The plasmid pJET-Rv1324m was extracted and confirmed through DNA sequencing. The Rv1324m was then subcloned into the expression vector pColdII, thereby yielding pColdII-Rv1324m. The expression conditions of Rv1324m in the BL21(DE3) cells and its purification and analyses were the same as those for Rv1324. The antioxidant activity of the Rv1324m protein was determined by a comparison to that of the Rv1324 protein.

### Infection of C57BL/6J mice with Rv1324/Msm and inhibitor assay.

The C57BL/6J mice were challenged with Rv1324/Msm using an aerosol generator (Kangjie Instrument, China). The control mice were challenged with Vec/Msm or PBST (PBS with 0.05% Tween 80). Bacterial cells suspended in PBST were aerosolized and delivered to each animal at 5 × 10^9^ CFU every day, and the infection lasted for 4 days.

To understand the role of ferroptosis in the Rv1324/Msm-infected mice, the mice in the Fer-1 groups were administered Fer-1 (Ferrostatin-1, an inhibitor of ferroptosis) at 3 mg/kg via intravenous injection once a day for 3 consecutive days before being infected with Rv1324/Msm or PBS.

Finally, the bacterial burden in the lung, the histopathology of the lung, and the levels of serum cytokines and ferroptosis in the lung were observed or determined. Three mice from each individual group at each time point were sacrificed and dissected.

**(i) Assessment of the colonization of Rv1324/Msm in mouse lungs.** On days 1, 4, 7, 14, and 21 postinfection, three mice from each group were sacrificed. The whole right lobe of the lung from each animal was obtained sterilely and homogenized in PBS buffer. The homogenates were serially diluted and plated on 7H11 agar plates. The bacterial burden was determined by counting the CFU after incubation for 3 days at 37°C.

**(ii) Pathology and histopathology analyses.** The left upper lobe of the lung from each mouse was obtained and fixed in 4% PFA for 24 h at 4°C. Then, the tissues were dehydrated, embedded in paraffin, and sectioned at a 3 μm thickness on a rotary microtome. After the slides were stained with hematoxylin and eosin (H&E), the pathological alterations of the lung tissues were observed under a Nikon Ni-U microscope (Nikon, Japan).

**(iii) Quantification of inflammatory factor levels.** On days 1, 4, 7, 14, and 21 postinfection, the levels of the serum cytokines TNF-α, IL-1β, and IL-6 were determined using Mouse ELISA Kits (Jiangsu Meibiao Biotechnology, China), according to the manufacturer’s instructions. Briefly, the sera of mice were collected via centrifugation at 2000 × *g* for 10 min. Then, 10 μL serum and 40 μL sample diluent were added to the appropriate wells and incubated at 37°C for 30 min. Then, the liquid was decanted, the biotinylated detection antibody working solution was added to the wells, and the mixture was incubated at 37°C for 60 min. The wells were washed 3 times with washing buffer, the HRP conjugate working solution was added, and the wells were incubated at 37°C for 30 min. Then, the wells were washed 5 times with washing buffer, and the substrate reagent was added for 15 min. The stop solution was added to the wells, and the absorbance at 450 nm was determined using a microplate spectrophotometer (MultiskanFC, Thermo Scientific).

**(iv) Measurement of the biomarkers of ferroptosis.** On day 4 postinfection, the left lower lobe of the lung was homogenized with PBS. The supernatant of the pulmonary homogenates was collected via centrifugation at 5000 × *g* for 10 min at 4°C. The levels of ROS and the biomarkers of ferroptosis, Fe^2+^, malondialdehyde (MDA), glutathione (GSH), and glutathione peroxidase 4 (GPX4) activity in the lung tissues were assessed.

The levels of ROS and GPX4 were assessed using a Mouse ROS ELISA Kit and a GPX4 ELISA Kit (Shanghai Enzyme-linked Biotechnology, China), respectively. The process is described above, under “Quantification of inflammatory factor levels”.

Intracellular ferrous and ferric ions were measured using an Iron Assay Kit (Solarbio, China). Briefly, the lung tissue was homogenized with tissue lysis buffer on ice. Then, the supernatant of the pulmonary homogenates was collected via centrifugation at 4,000 × *g* and 4°C for 10 min. After adding 180 μL of iron assay buffer and 120 μL of sample to a 1.5 mL tube, the mixture was placed in boiling water for 5 min. Then, 60 μL of chloroform were added to the mixture, and the mixture was vortexed. 200 μL of supernatant from each tube were collected via centrifugation at 10,000 × *g* and 4°C for 10 min and were then transferred to a 96-well plate. The absorbance at 520 nm was detected. In addition, the standard curve was generated, according to the manufacturer’s instructions.

The lipid peroxidation was evaluated using an MDA Assay Kit (Beyotime, China), according to the manufacturer’s instructions. Briefly, 100 μL of the supernatant of the pulmonary homogenates were mixed with 200 μL malondialdehyde solution in a 1.5 mL tube and incubated for 15 min at 100°C in the dark. After all of the mixtures cooled to room temperature, they were centrifuged at 1000 × *g* and 25°C for 10 min. 200 μL of supernatant from each tube were transferred to a 96-well plate, and the absorbance at 532 nm was determined. A standard curve was generated, according to the manufacturer’s instructions.

The intracellular total GSH was measured using a GSH Assay Kit (Beyotime, China), as described by the manufacturer. Briefly, the supernatant of the pulmonary homogenates was diluted with protein removing buffer S (1:4) and centrifuged at 10,000 × *g* for 10 min at 4°C for collection. 10 μL of the supernatant were mixed with 150 μL GSH working solution and 50 μL 0.5 mg/mL NADPH in a 96-well plate. After mixing and vortexing, the GSH content was immediately measured via the determination of the absorbance at 412 nm. The standard curve was prepared, according to the manufacturer’s instructions.

### Statistical analysis.

All measurements are depicted as the mean ± SEM obtained from three independent experiments to correct the trial errors. The differences between groups were analyzed using a one-way or two-way analysis of variance (ANOVA), and this was followed by Bonferroni’s multiple-comparison test. The GraphPad Prism 9 software package (version 9.0, GraphPad, San Diego, CA, USA) was used to carry out the analyses. A *P* value of <0.05 is considered to be indicative of a statistically significant result. *, *P* < 0.05; **, *P* < 0.01; ***, *P* < 0.001.
