# Supplementary material for: Mycobacterium tuberculosis Rv1324 Protein Contributes to Mycobacterial Persistence and Causes Pathological Lung Injury in Mice by Inducing Ferroptosis
Source: Microbiol Spectr. 2023 Jan 10;11(1):e02526-22. doi: 10.1128/spectrum.02526-22 (PMC9927160; doi:10.1128/spectrum.02526-22)

Supplementary Materials for

Manuscript: "*Mycobacterium tuberculosis* Rv1324 protein contributes to mycobacterial persistence and causes pathological lung injury in mice via inducing ferroptosis"

This file includes:

Supplementary figure S1 to S4

Supplemental Fig. S1. Identification of the purified Rv1324 by MALDI-TOF MS.

The peptides detected by MALDI-TOS MS were matched to Rv1324 of *Mycobacterium tuberculosis* H37Rv.

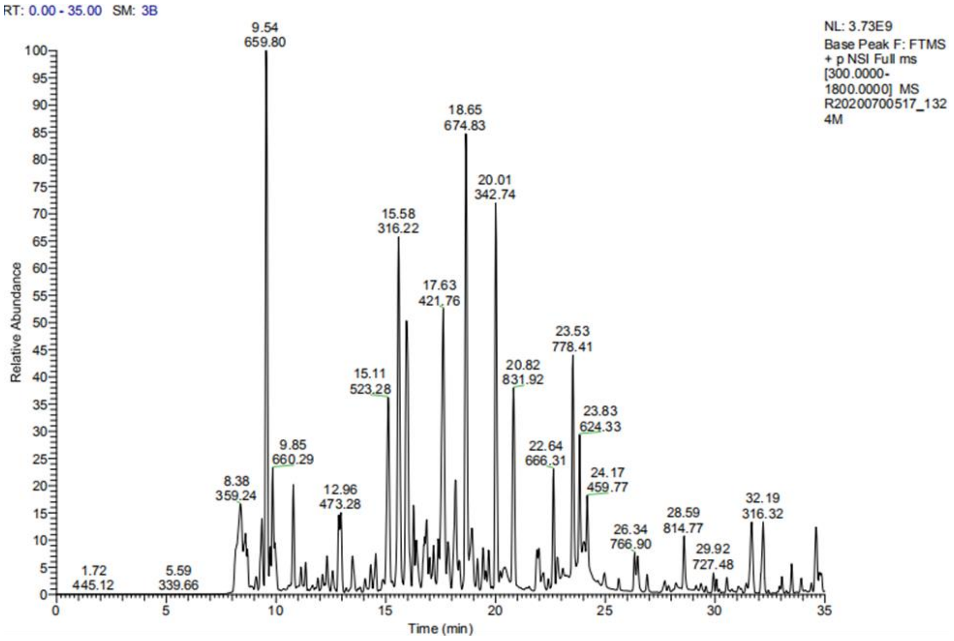

Sequence coverage:58.36%

Matched peptide shown in Bold Red

1 MTRPRPPLGPAM**AGAVDLSGIK**QRAQQNAAASTDADRALSTPSGVTEITE

51 ANFEDEVIVRSDEVPVVVLLWSPR**SEVCVDLLDTLSGLAAAAK**GK**WSLAS**

101 **VNVDVAPR**VAQIFGVQAVPTVVALAAGQPISSFQGLQPADQLSR**WVDSLL**

151 **SATAGKLKGAASSEESTEVDPAVAQARQQLEDGDFVAARKSYQAILDANP**

201 **GSVEAK**AAIR**QIEFLIR**ATAQRPDAVSVADSLSDDIDAAFAAADVQVLNQ

251 DVSAAFERLIALVRRTSGEERTRVRTR**LIELFELFDPADPEVVAGR**R**NLA**

301 **NALY**

Supplemental Fig. S2. Multiple sequence alignment of Mtb Trxs and Mtb Rv1324. The sequence alignment of TrxA, TrxB, TrxC and Rv1324 was generated with CLC Protein Workbench 2.0.2. "Cys-X-X-Cys" motif shown in the red box is highly conserved in the Trxs superfamily but "Ser_{75}-X-X-Cys_{78}" motif is present in Rv1324 protein. A histogram showed amino acid conservation among Trxs and Rv1324.

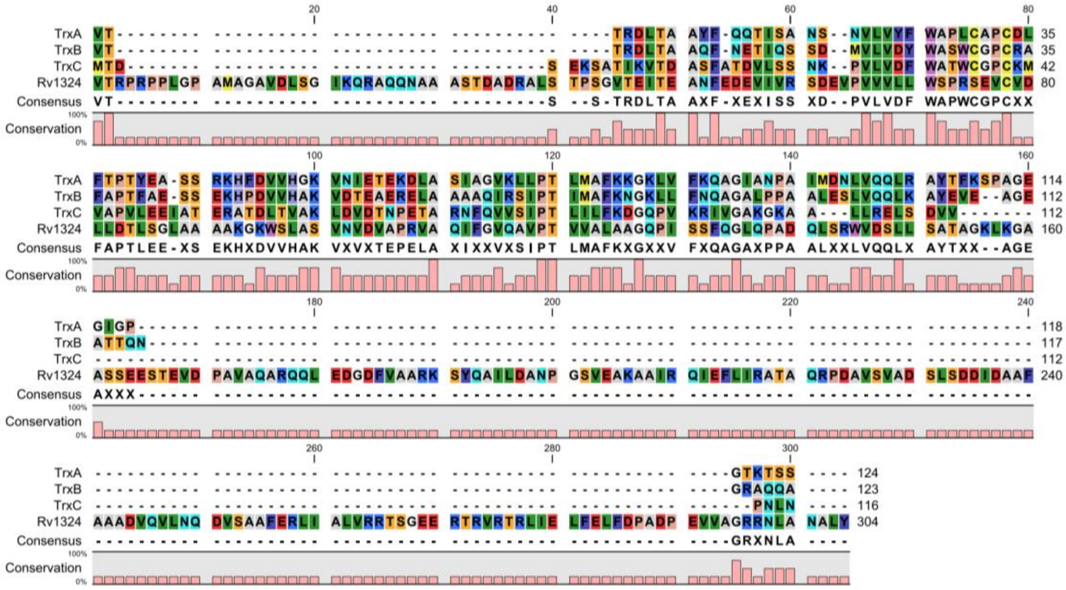

Supplemental Fig. S3. Identification of Rv1324 activity to reduce disulfide bonds in insulin. *M. tuberculosis* TrxC was also constructed and over-expressed in the *E. coli* BL21(DE3) strain. Then, TrxC was purified by a Ni-NTA SuperFlow resin. The purified TrxC protein was as a control protein. To detect the activity of reduce disulfide bonds, an insulin reduction assay was preformed according to the previously described （1）. Briefly, 100 μL reaction mixture contained 100 mM phosphate buffered saline (PBS), 1.25 mg/mL bovine insulin, and 0.15 mg/mL purified protein (Rv1324 or TrxC). The reaction was initiated by the addition of 4 μL of 10 mM dithiothreitol (DTT). The purified protein without DTT was used as negative control. The absorbance at 650 nm was measured by a microplate spectrophotometer (Multiskan FC, Thermo Scientific). The increase in turbidity accompanying reduction of the disulfide bonds of insulin was monitored.

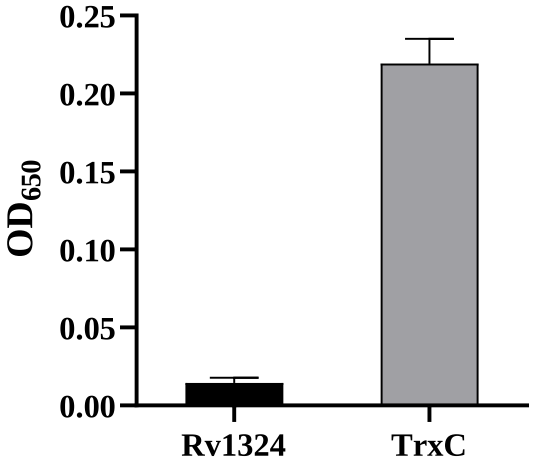

References

1. Luthman M, Holmgren A. 1982. Rat liver thioredoxin and thioredoxin reductase: purification and characterization. Biochemistry 21(26):6628-33.

Supplemental Fig. S4. Detection of Rv1324m expression in pCold-Rv1324m/*E. coli* BL21(DE3) by SDS-PAGE (A) and Western blotting (B) analyses. The supernatant of pCold-Rv1324m/*E. coli* BL21(DE3) and *E. coli* BL21(DE3) strains were collected and lysated. Twenty micrograms of whole-cell lysate proteins was added to SDS-PAGE gels for analyzing Rv1324m protein expression. For western blotting analysis, the proteins bands were visualized in NBT/BCIP solution. Lane 1 represents the proteins expressed in the supernatant of *E. coli* BL21(DE3) strain, lanes 2 represents the protein expressed in the supernatant of pCold-Rv1324m/*E. coli* BL21(DE3) and lane M represents the PageRuler pre-stained protein ladder.

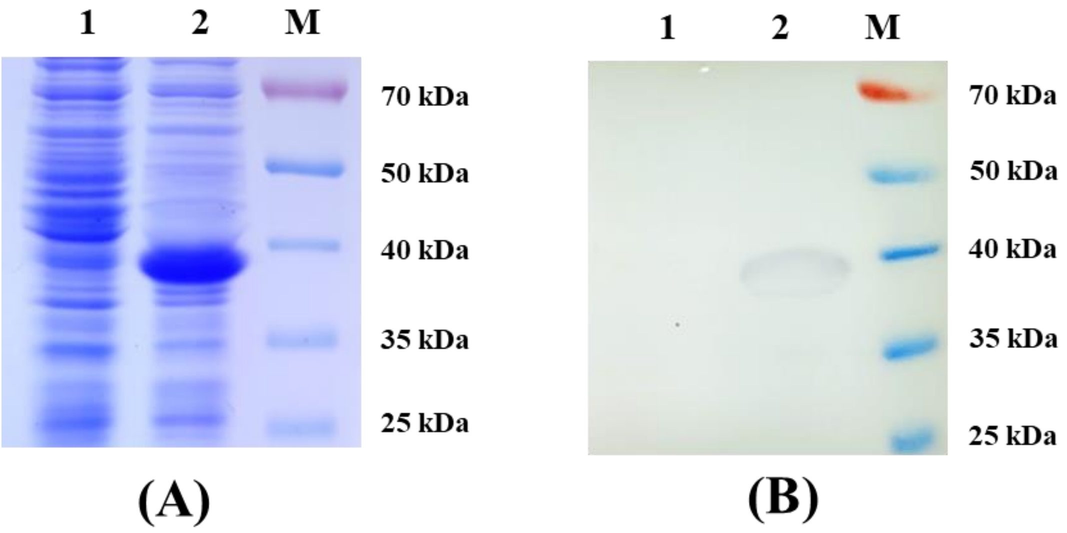

Supplement: Supplemental file 1 — Fig. S1 to S4. Download spectrum.02526-22-s0001.pdf, PDF file, 0.4 MB [file spectrum.02526-22-s0001.pdf]
